# A simple microfluidic device for live-cell imaging of *Arabidopsis* cotyledons, leaves, and seedlings

**DOI:** 10.2144/btn-2018-0044

**Published:** 2018-06

**Authors:** Shia Vang, Kati Seitz, Patrick J Krysan

**Affiliations:** 1 Horticulture Department, University of Wisconsin-Madison, 1575 Linden Drive, Madison, WI 53706, USA;; 2 Laboratory of Genetics, University of Wisconsin-Madison, Madison, WI, USA;; 3 Genome Center of Wisconsin, University of Wisconsin-Madison, Madison, WI, USA

**Keywords:** *Arabidopsis*, live-cell imaging, microfluidic, microscopy, sample mounting

## Abstract

One of the challenges of performing live-cell imaging in plants is establishing a system for securing the sample during imaging that allows for the rapid addition of treatments. Here we report how a commercially available device called a HybriWell™ can be repurposed to create an imaging chamber suitable for *Arabidopsis* seedlings, cotyledons and leaves. Liquid in the imaging chamber can be rapidly exchanged to introduce chemical treatments via microfluidic passive pumping. When used in conjunction with fluorescent biosensors, this system can facilitate live-cell imaging studies of signal transduction pathways triggered by different treatments. As a demonstration, we show how the HybriWell can be used to monitor flg22-induced calcium transients using the R-GECO1 calcium indicator in detached *Arabidopsis* leaves.

## Introduction

Live-cell imaging refers to an experimental approach whereby living samples are observed via microscopy in order to study a biological process at the cellular and/or subcellular level. This approach stands in contrast to traditional microscopy in which non-living, fixed samples are analyzed. Live-cell imaging can be used to study cell biology, signal transduction, and a range of other biological phenomena. An important consideration for these experiments is that one needs to have a method available for mounting the sample that keeps it viable and in a stress-free condition throughout the imaging process. In addition, if one wishes to study how the sample responds to a chemical stimulus, the mounting method needs to allow for pulses of chemical treatments to be easily added and removed from the imaging chamber with minimal collateral stress.

Most live-cell imaging studies involve the use of fluorescent probes and reporters in conjunction with epifluorescent or confocal microscopy. In the case of plant biology, a number of fluorescent sensors have been developed that allow one to measure the concentrations of glucose, inorganic phosphate, zinc, ATP, hydrogen peroxide, pH, and calcium in living plant tissue, and fluorescent reporters for the plant hormones auxin, gibberellic acid (GA), and abscisic acid (ABA) have also been described [1–12]. A recent review summarizes the current state-of-the-art in plant live-cell imaging and biosensors [13]. In addition, fusion proteins tagged with fluorescent reporters allow one to track the subcellular localization of a protein of interest and monitor its interactions with other proteins [14]. By using live-cell imaging methods, one can observe these factors in real time and also determine how the sample responds to a stimulus.

An elegant method for performing live-cell imaging studies of *Arabidopsis* root tissue has been previously described. This system, called the RootChip, is based on a custom-built microfluidic device that channels the growing primary root of an *Arabidopsis* seedling into a microfluidic channel fabricated on the surface of a microscope cover glass [1, 15, 16]. The microfluidic channels allow one to flow pulses of chemical treatments across the root tissue while the sample is mounted on a microscope for imaging. This strategy has been used to study a range of biochemical and signaling processes in *Arabidopsis* roots [1, 4, 5, 15, 16].

In the case of studies focused on leaf tissue, a variety of custom imaging chambers have been described that aim to hold the sample in place during microscopy while allowing chemical treatments to be perfused into the chamber [17–19]. Although effective for imaging experiments, these custom chambers can be challenging to replicate by other labs. We were therefore interested in developing a simple, reproducible method for mounting *Arabidopsis* leaves for live-cell imaging that could be easily adopted by other labs with minimal effort. Here we describe how a commercially available device called a HybriWell™ (Grace Bio-Labs, OR, USA), which is designed for performing microarray hybridization experiments, can be repurposed to serve as a live-cell imaging chamber for *Arabidopsis* leaves and seedlings.

## Materials & methods

### Chemicals & supplies

Propidium iodide (PI) solution (1 mg/ml in water) was obtained from Sigma-Aldrich (MO, USA; Cat. # P4864). For flg22 treatment, synthetic flg22 peptide (QRLSTG-SRINSAKDDAAGLQIA) was obtained from PhytoTechnology Laboratories (Shawnee Mission, KS, USA; Cat. # P6622), and the flg22 was dissolved in water to a concentration of 1 μM. HBW20 HybriWell devices were obtained from Grace Bio-labs (Cat. # 611102). 45 × 50 mm microscope cover glasses 0.16–0.19 mm thick were obtained from Fisher Scientific (MA, USA; Cat. # 12–544F).

### Plant materials & growth conditions

*Arabidopsis thaliana* seedlings were grown on 1% agar (w/v) plates containing containing 0.5 × Murashige and Skoog (MS) basal salt mixture under constant light at a light intensity of 115–130 μmol/m ^2^ sec at 20–23°C with the plates in a vertical orientation. A transgenic line expressing the intensiometric calcium indicator R-GECO1 was a kind gift from Melanie Krebs, and is described as line #7–8 from Keinath *et al.* [17]. Experiments with detached cotyledons used seedlings that were 4–6 days old. Experiments with intact seedlings used 3-day-old seedlings. For experiments using detached true leaves, seedlings were first germinated on agar plates and then transplanted to soil after 1 week. Leaf samples were then collected from 2-week-old plants in soil.

### Mounting samples in the HybriWell™

To mount an *Arabidopsis* cotyledon in a HybriWell, a 45 × 50 mm microscope cover glass was laid on a lab bench. A 5-μl drop of sterile ultrapure water was then placed in the center of the cover glass using a micropipette. Next, a cotyledon was removed from a 4- to 6-day-old seedling growing on a 0.5 × MS plate by snipping the petiole with small scissors. The cotyledon was grasped by the petiole using forceps and gently placed on the water drop with the abaxial surface facing up. Since the cotyledon typically floats on the surface of the water drop, a micropipette was then used to transfer water from below the cotyledon onto the top surface in order to fully wet the cotyledon on both its abaxial and adaxial surfaces. Excess water was then removed using the micropipette, being careful not to damage the cotyledon. The adhesive backing on the HybriWell was then exposed by peeling off the protective covering and forceps were used to gently lower the HybriWell down onto the cover-glass, positioning it such that the cotyledon is in the center of the HybriWell chamber. The back of the forceps is then used to gently press the adhesive portion of the HybriWell down onto the cover glass to fully seal the chamber, taking care not to press down on the top of the chamber. To fill the chamber with water, 300 μl of sterile ultrapure water was rapidly dispensed into one of the ports of the HybriWell such that water shoots out the second port during the filling process. This approach is necessary in order to minimize the chance of trapping an air bubble in the chamber. If an air bubble is trapped in the chamber, then an additional 300 μl of sterile ultrapure water can be flushed through the HybriWell to remove the bubble. Finally, a 200 μl drop of sterile ultrapure water was placed on top of one of the ports of the HybriWell to prevent evaporation of the 30 μl of water within the chamber. The HybriWell mounted to the cover glass was then placed inside a 100 × 15 mm round plastic Petri dish and covered to maintain sterility and humidity.

The same process described above was used to mount intact seedlings, but in that case the seedlings were transferred to the drop of water on the cover glass by gently lifting them off the growth plate with a sterile plastic 200-μl pipette tip. For mounting true leaves, a single leaf was collected from a plant growing in soil by snipping the petiole with scissors and grasping the detached leaf by the petiole using forceps. The same process described above for cotyledons was then used to mount the true leaf samples in the HybriWell.

### Imaging

Samples were mounted as described above and then incubated in the HybriWell for 6–8 h prior to imaging. During this time the samples were maintained under constant light at 20–23°C at a light intensity of 115–130 μmol/m ^2^ sec.

For the experiments using PI and green fluorescent protein (GFP), images were collected by confocal microscopy using a Zeiss (Oberkocha, Germany) LSM 710 with 10x and 20x objectives. PI fluorescence was imaged by laser excitation at 594 nm with emission collected between 600 and 643 nm. GFP fluorescence was imaged by laser excitation at 488 nm with emission collected between 500 and 541 nm. 3-μm optical sections were collected as a Z-stack covering a total distance of 63 μm. Images were analyzed using Fiji software.

For experiments using R-GECO1, images were collected by confocal microscopy using a Zeiss LSM 780 with a 20x objective. Samples were placed on the microscope and allowed to equilibrate for 30 min prior to imaging. R-GECO1 fluorescence was imaged by laser excitation at 561 nm with emission collected between 579 and 651 nm and an optical slice thickness of 2 μm. Single images were collected every 5 secs for the duration of the experiment. Images were analyzing using Fiji software, and the Fire lookup table was used to false-color the image to reflect fluorescent signal intensity.

### Root growth rate measurement

*Arabidopsis* Col-0 seeds were surface-sterilized and stratified on a 0.5 × MS plate at 4°C for 2 days. The plate was then placed vertically under constant light at 20–23°C for 3 days. Six seedlings were then mounted in sterile water in HybriWells, one seedling per HybriWell. Another six seedlings were transferred to a new 0.5 × MS plate. The HybriWells were incubated horizontally in covered Petri dishes under constant light at 20–23°C. The 0.5 × MS plates were incubated in a vertical orientation. Images of the seedlings were captured at 0- and 6-h time points using an Epson Perfection V700 Photo Scanner at 4800 dpi resolution (Epson, Nagano Prefecture, Japan). Root length was determined using Fiji software and growth rate was calculated.

## Results & discussion

### Repurposing the HybriWell for live-cell imaging in *Arabidopsis*

**Figure 1.**
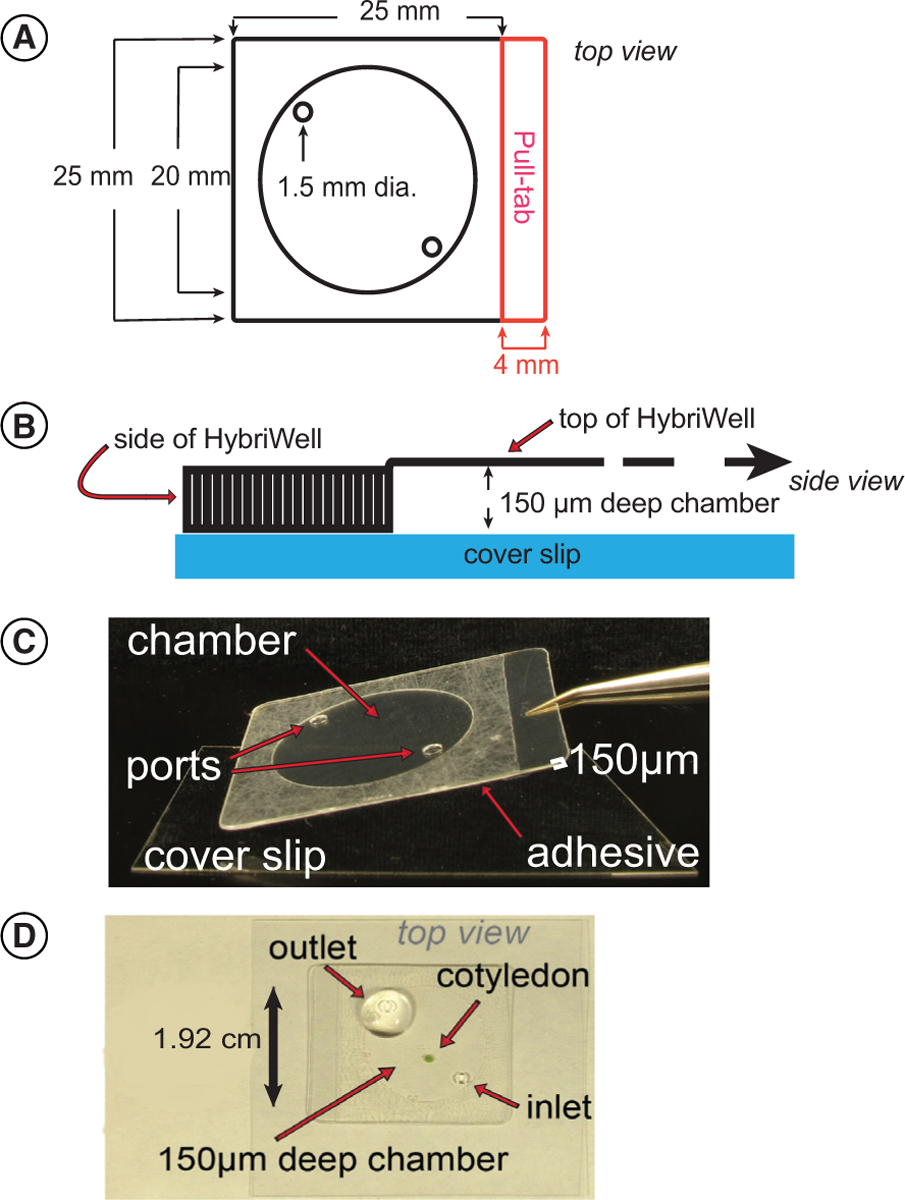
Overview of the HybriWell™ device. **(A)** Top view schematic of the HybriWell. This device consists of a small chamber with two ports. The bottom layer is adhesive. Red indicates the non-adhesive peel tab. **(B)** Side view schematic of the HybriWell system. The HybriWell is laid on top of a cover glass, creating a sealed chamber that can be filled with water to hold a sample for imaging. The striped lines represent the adhesive outer area. **(C)** Image of a HybriWell about to be laid on top of a microscope cover glass. **(D)** Image of a HybriWell attached to a microscope slide and containing an *Arabidopsis* cotyledon sample. The chamber is filled with water and a large droplet is placed on the ‘outlet’ port. The cotyledon can be imaged through the cover glass using an inverted microscope.

The HybriWell is a disposable plastic device that was originally designed to create low-volume chambers on microscope slides for use in microarray hybridization experiments. HybriWells are available in a variety of sizes and shapes, but the one that we selected for our experiments is a square patch of plastic 25 by 25 mm in size with a 20-mm diameter circular chamber in the center (Figure 1A). After peeling the protective cover off the adhesive side of the HybriWell, one can press the HybriWell down onto the surface of a microscope cover glass to form a chamber 20 mm in diameter and 150-μm deep (Figure 1B). The resulting chamber has an approximate volume of 30 μl. The top of the chamber is clear plastic, and the bottom is formed by the glass cover glass. The sides of the HybriWell are held tight to the cover glass by the adhesive. On top of the chamber there are two 1.5-mm diameter ports that are used to introduce liquid into the chamber once it has been mounted on the cover glass.

When using the HybriWell to image *Arabidopsis* samples, one first places the sample in a 5-μl drop of water on a glass cover glass. Next, a HybriWell with the adhesive exposed is carefully laid over the top of the sample using forceps (Figure 1C). Finally, a pipette is used to force water into one of the ports on top of the HybriWell, thus filling the HybriWell with liquid. The sample is gently held in place between the clear plastic top of the HybriWell and the cover glass. We have found that the 150 μm depth of the chamber works well for mounting detached *Arabidopsis* cotyledons and leaves as well as young seedlings up to a few days old. Since the sample is mounted on top of a microscope cover glass, imaging can be performed using an inverted microscope. As the adhesive material on the HybriWell forms a strong bond to the cover glass it is not practical to remove the HybriWell from the cover glass once a sample has been mounted. The HybriWell is therefore a disposable device that can only be used to analyze one sample before discarding it.

### Passive pumping can exchange liquid within the HybriWell

After determining that the depth of the HybriWell chamber was suitable for mounting *Arabidopsis* samples, we next wanted to test how effectively the liquid in the chamber could be exchanged with fresh solution. Since our ultimate goal is to be able to apply chemical treatments to samples during live-cell imaging, it is critical that the liquid within the HybriWell can be efficiently exchanged. Fluid movement through the HybriWell device can be driven by the microfluidic process of passive pumping [20]. To achieve passive pumping, one first places a large drop of water over one of the 1.5 mm ports on top of the HybriWell (Figure 1D). If liquid is then applied dropwise to the other port, it will be rapidly drawn into the HybriWell by passive pumping. Each time a drop of liquid is added to the inlet port, it quickly enters the HybriWell and an equal volume of liquid exits the HybriWell into the growing drop covering the outlet port. It is important to stress that one does not use positive pressure to force liquid into the inlet port rather, a drop of liquid is gently dropped into the inlet port, and passive pumping pulls that liquid into the HybriWell.

**Figure 2.**
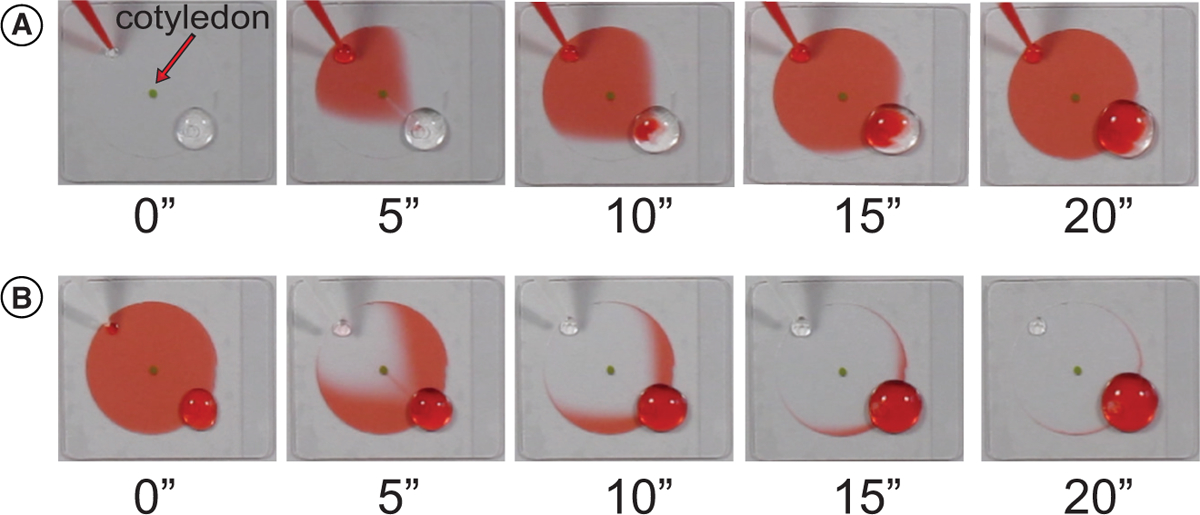
Time-lapse demonstration of HybriWell™ fluidics. **(A)** 50 μl of red food coloring was added in small droplets to the ‘inlet’ port of a HybriWell mounted on a coverglass. The HybriWell contained an *Arabidopsis* cotyledon and was filled with clear water prior to adding the food coloring. Through the process of passive pumping, the water within the HybriWell was rapidly replaced with the red food coloring. **(B)** The process was repeated by adding 50 μl of water containing no food coloring.

In order to visualize the movement of liquid through the HybriWell chamber, we first mounted a detached *Arabidopsis* cotyledon in water using a HybriWell as described above. A 50-μl drop of water was placed over the outlet port, and then 50 μl of red food coloring solution was introduced dropwise into the inlet port. At time zero, the small green cotyledon can be seen in the center of the clear water in the HybriWell chamber. In as little as 5 sec, the red food coloring solution has surrounded the cotyledon, and by 20 sec it has filled the chamber (Figure 2A and Supplemental Video 1). To demonstrate the ability to perform pulse-chase experiments using the HybriWell, we next added 50 μl of pure water to the chamber that had been filled with red food coloring (Figure 2B and Supplemental Video 2). The clear water quickly replaced the red food coloring in the chamber. It should be noted that when liquid is introduced into the HybriWell by passive pumping, there is an interface between the new and old solutions that sweeps across the chamber. Since the chamber volume is ~ 30 μl, adding 50 μl of new solution effectively replaces the old solution in the chamber with the new solution. The approximate flow rate with which the front of red food coloring sweeps across the HybriWell in Supplemental Video 1 is 3 mm/sec.

Next we wanted to determine how the HybriWell affected seedling growth. For this experiment 3-day-old seedlings were mounted in HybriWells, and root length was measured at 0 and 6 h after mounting. For comparison, control seedlings growing on the surface of agar growth media plates were also measured. We determined that the average growth rate of primary roots in HybriWells was 0.38 mm/h ± 0.09 mm/h (n = 6) versus 0.44 mm/h ± 0.03 mm/h (n = 6) for the control samples. This difference was not statistically significant (two-tailed t-test; p = 0.19).

### Epidermal cells are exposed to chemical treatments added to the HybriWell

The experiments using red food coloring described above demonstrated that the liquid within the HybriWell chamber can be efficiently exchanged. When an *Arabidopsis* sample is mounted in a HybriWell for imaging, it is sandwiched between the top of the HybriWell and the glass cover. If the HybriWell is to be used for live-cell imaging experiments, it is important that a chemical treatment introduced into the HybriWell is able to rapidly reach the surface of the tissue that is being imaged. We therefore needed to determine if liquid added to a HybriWell through the inlet port efficiently flows through the space between the cotyledon and the glass cover. If the sample were too tightly pressed down upon the cover glass, it might prevent the added treatment from contacting the epidermis.

**Figure 3.**
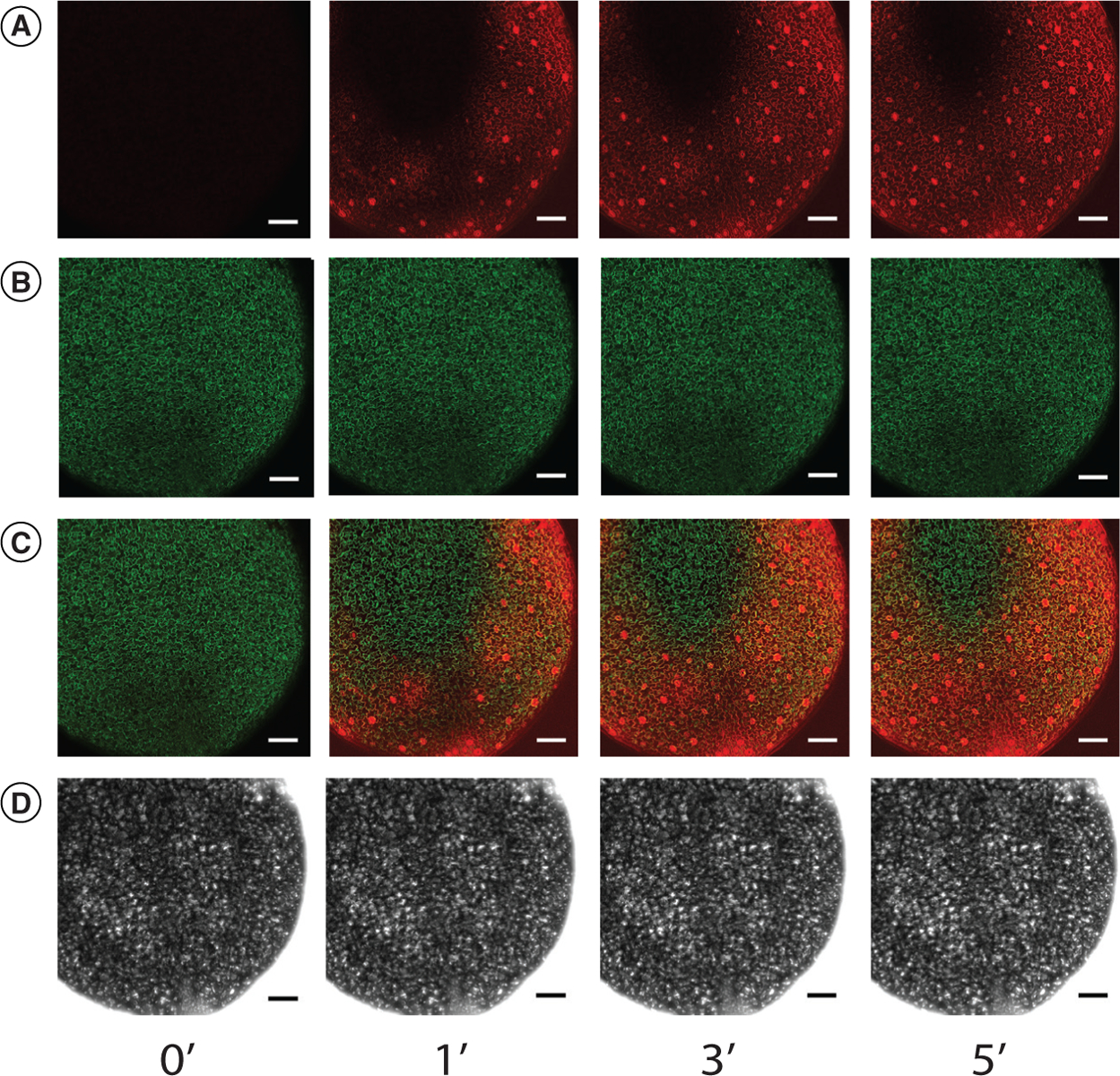
Propidium iodide staining of cotyledons in the HybriWell™. An *Arabidopsis* cotyledon expressing 35S:GFP was mounted in water in a HybriWell and treated with 50 μl of propidium iodide solution. Maximum Z-projections of confocal images collected before treatment (0’) and at the indicated time points in minutes after treatment are shown. **(A)** Propidium iodide channel. **(B)** GFP channel. **(C)** Merged images. **(D)** Brightfield images. Scale bar = 100 μm.

To address this question, we mounted a cotyledon in water as described above. The sample was then placed on an inverted confocal microscope such that the lens pointed up through the coverglass to the epidermis of the cotyledon. Next, 50 μl of 300-μM propidium iodide (PI) was added to the HybriWell through the inlet port. PI is a red fluorescent dye that has been previously shown to stain plant cell walls. In addition, the *Arabidopsis* seedlings used for this experiment expressed GFP throughout the cotyledon, which allowed us to visualize the cotyledon epidermis. An image collected 1 min after PI addition showed strong red fluorescence highlighting the cell walls of a substantial portion of the cotyledon epidermis, and the area of the epidermis stained by PI increased at the 3- and 5-min time points (Figure 3). The images shown in Figure 3 are maximum intensity projections of Z-stack series focused on the epidermal layer.

In these experiments we routinely observed that the red fluorescent signal in the central portion of the cotyledon was substantially lower than that observed in the outer regions (Figure 3A). These observations suggest that the flow of liquid through the HybriWell may be restricted in areas where the cotyledon is pressed tightly against the cover glass. Our observation that a majority of the sample becomes quickly stained, however, indicates that a substantial portion of the cotyledon epidermis is rapidly contacted by chemical treatments introduced into the HybriWell by passive pumping. These results suggest that there is a layer of liquid between the epidermis and the cover glass in this imaging setup that allows introduced chemical treatments to easily flow across the sample. It is important to recognize, however, that the entire cotyledon is not likely to be uniformly exposed to the treatment, with the inner portion of the sample less likely to be exposed. Focusing on regions of the epidermis near the margin of the cotyledon provides the best opportunity for exposure to the chemical treatment. In addition, a fluorescent dye could be used in conjunction with the treatment of interest to serve as a tracer for identifying cells that were directly exposed to the treatment.

We next wanted to determine if the HybriWell could be used to image *Arabidopsis* roots. For this experiment we mounted intact 3-day-old *Arabidopsis* seedlings in the HybriWell using the same technique described above for cotyledons. 1 min after adding 50 μl of 300-μM PI to the HybriWell we observed strong PI staining of the entire root (Supplemental Figure 1). The fluorescent signal did not increase in intensity when images were collected at the 3- and 5-min time points. These results indicate that the entire root is rapidly exposed to the PI treatment delivered via the HybriWell.

### Imaging flg22-induced Ca^2+^ transients using the R-GECO1 calcium indicator

**Figure 4.**
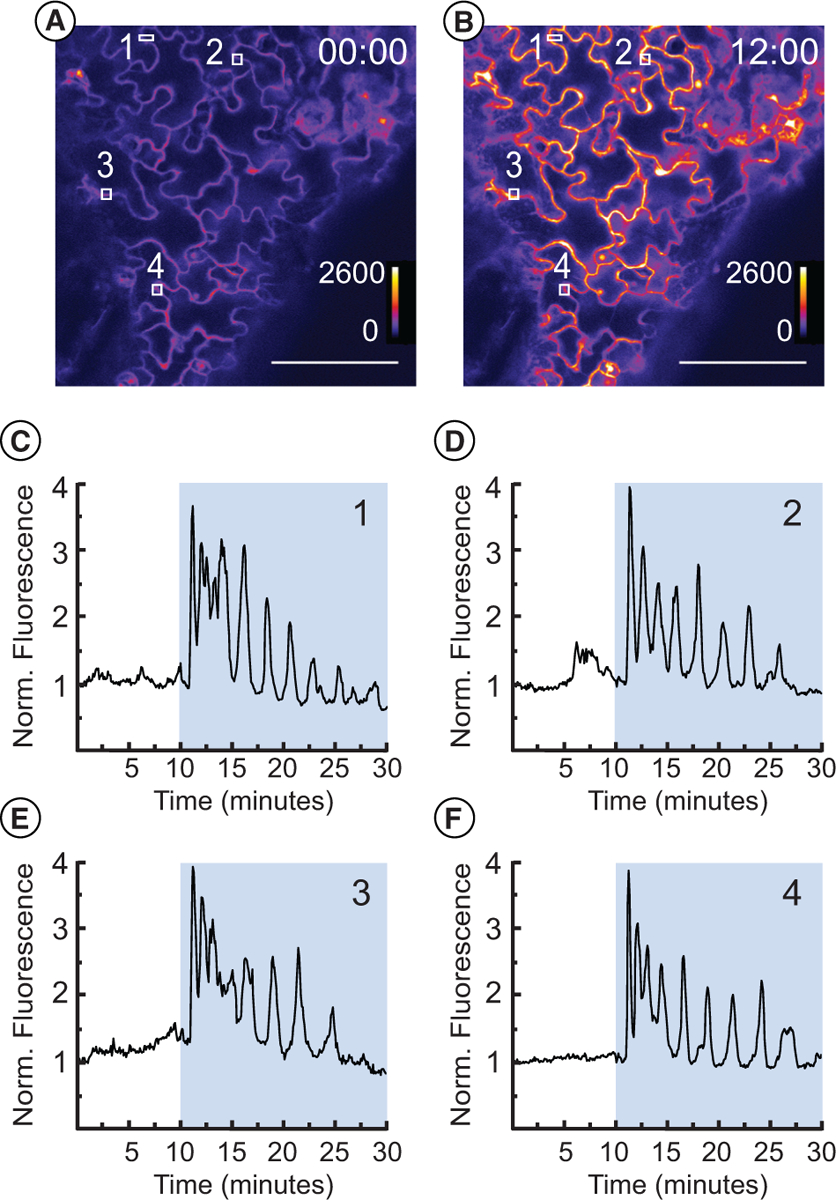
Calcium imaging with R-GECO1 using a HybriWell™ in response to flg22. An *Arabidopsis* true leaf expressing the intensiometric calcium indicator R-GECO1 was mounted in water in a HybriWell and imaged before and after treatment with 100 nM flg22. Images of the leaf epidermis were collected every 5 secs using a confocal microscope. **(A)** Leaf epidermis prior to treatment. **(B)** Leaf epidermis 115 seconds after treatment. Fluorescence intensity of the R-GECO1 signal in arbitrary units from 0 to 2600 is shown using a heat map scale. Time stamp indicates minutes:seconds. Scale bar = 100 μm. White boxes indicate the regions of interest used in C-F. **(C-F)** Normalized R-GECO1 fluorescence for individual regions of interest. Number in the upper right of each graph indicates the corresponding regions of interest. Blue background indicates presence of 100 nM flg22 in the HybriWell.

Fluorescent biosensors that report the concentration or activity of signal transduction molecules are valuable tools for live-cell imaging studies. For example, R-GECO1 is an intensiometric calcium indicator in which the intensity of red fluorescence is proportional to free calcium concentration [3,17,21]. In order to evaluate the utility of the HybriWell for studying signal transduction using fluorescent biosensors, we tested the response of true leaves expressing the R-GECO1 calcium indicator to the bacterial elicitor peptide flg22. flg22 is a conserved sequence from bacterial flagella that has been shown to cause rapid calcium spiking in *Arabidopsis* [17,22,23]. We mounted true leaves expressing R-GECO1 in HybriWells and collected images every 5 secs using a confocal microscope in order to quantify the intensity of red fluorescent emission. Addition of 100 μM flg22 to the HybriWell via passive pumping caused a sharp gain in red fluorescence ~ 1 min after treatment, indicative of a rapid increase in free calcium (Figure 4 and Supplemental Video 3). Analysis of four different regions of interest in the images revealed similar oscillating patterns of red fluorescence, consistent with previously reported experiments involving flg22 treatment of *Arabidopsis* leaves expressing R-GECO1 [17]. In order to evaluate the reproducibility of the HybriWell system for performing live-cell imaging studies, we repeated this flg22 experiment two additional times and observed similar results: a rapid gain in red fluorescence 1 to 2 min after flg22 treatment followed by oscillations in fluorescence intensity (Supplemental Figure 2 and Supplemental Videos 4 and 5).

Because calcium signaling is known to be sensitive to mechanical stimulation, we also performed a control experiment where pure water was added to the HybriWell, rather than flg22 (Supplemental Figure 3 and Supplemental Video 6). We did not observe any strong increase in red fluorescence upon adding pure water to the HybriWell, demonstrating that the process of passive pumping through the device does not produce any strong mechanical stimulation. This feature of the HybriWell suggests that it should be possible to use this device to test the effect of a chemical treatment without confounding effects caused by mechanical stress.

Taken together, our results demonstrate that the HybriWell device is able to function as a microfluidic chamber for performing live-cell imaging studies of *Arabidopsis* leaves and seedlings. We have observed that samples can be maintained in the HybriWell chambers for at least 6 h with no apparent negative effects on growth, indicating that this system is suitable for relatively long-term time-course experiments. Because HybriWell devices are low cost and commercially available, it should be straightforward for laboratories interested in performing live-cell imaging of *Arabidopsis* to adopt them for use in their experimental systems.

## Supplementary Material

Supplementary

## METHOD SUMMARY

We have developed a sample mounting chamber for performing live-cell imaging experiments of *Arabidopsis thaliana* by repurposing a commercially available device called the HybriWell™. This inexpensive, disposable device creates a chamber in which the liquid surrounding the living sample can be rapidly exchanged via microfluidic passive pumping.
