# Supplementary material for: A simple microfluidic device for live-cell imaging of Arabidopsis cotyledons, leaves, and seedlings
Source: Biotechniques. Author manuscript; Available in PMC 2021 Nov 15. (PMC8592504; doi:10.2144/btn-2018-0044)
Supplement: Supplementary [file NIHMS1631172-supplement-Supplementary.zip › Supplementary figures.pdf]

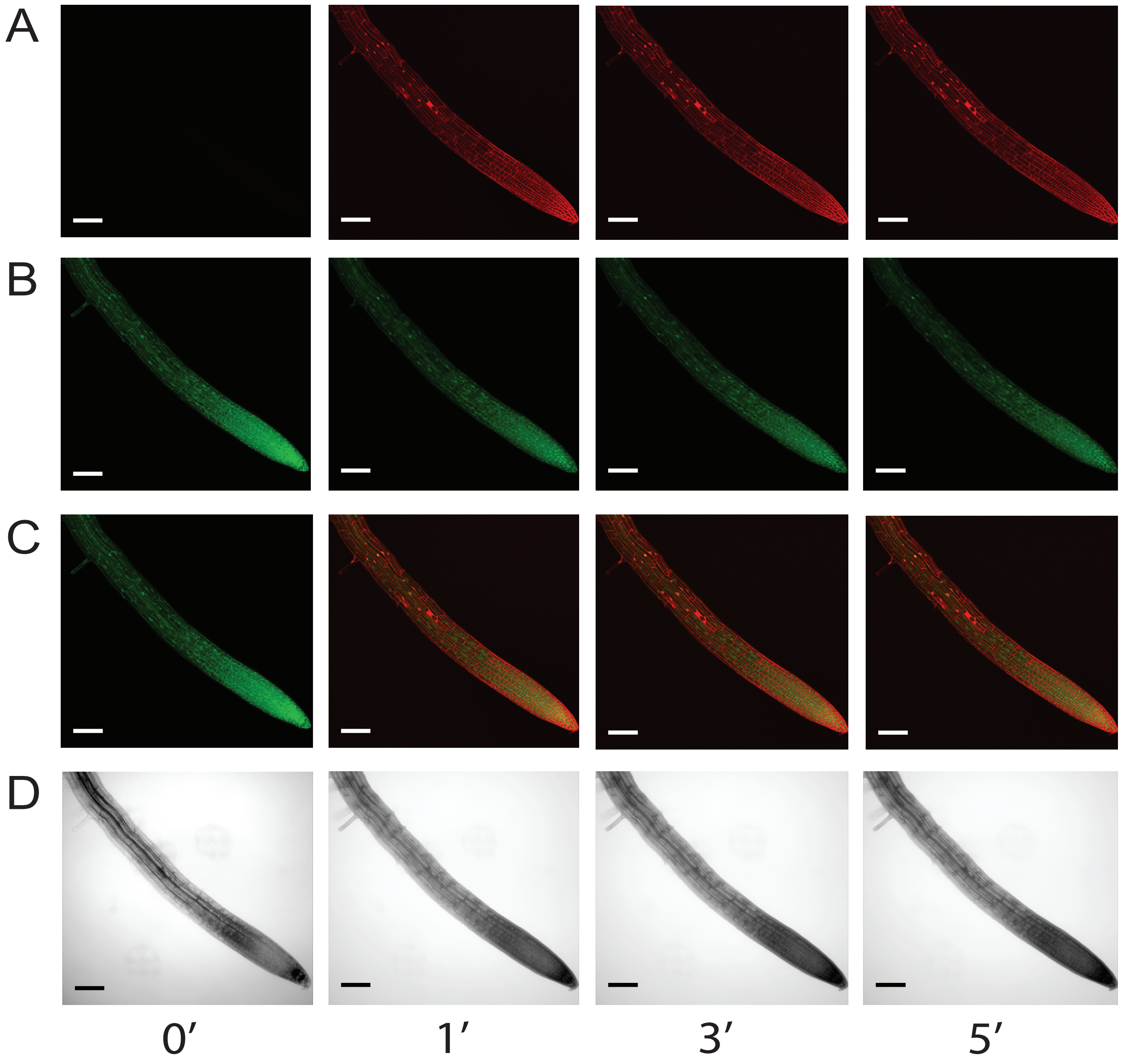

Supplemental Figure 1. Propidium iodide staining of roots in the Hybriwell.
Three-day-old seedlings expressing 35S:GFP were placed in Hybriwells, and the samples were treated with 50 μl of 33 μM propidium iodide. Maximum Z-projections of confocal images collected before treatment (0') and at the indicated time points in minutes after treatment are shown. (A) Propidium iodide channel. (B) GFP channel. (C) Merged images. (D) Brightfield images. Scale bar=100 μm.

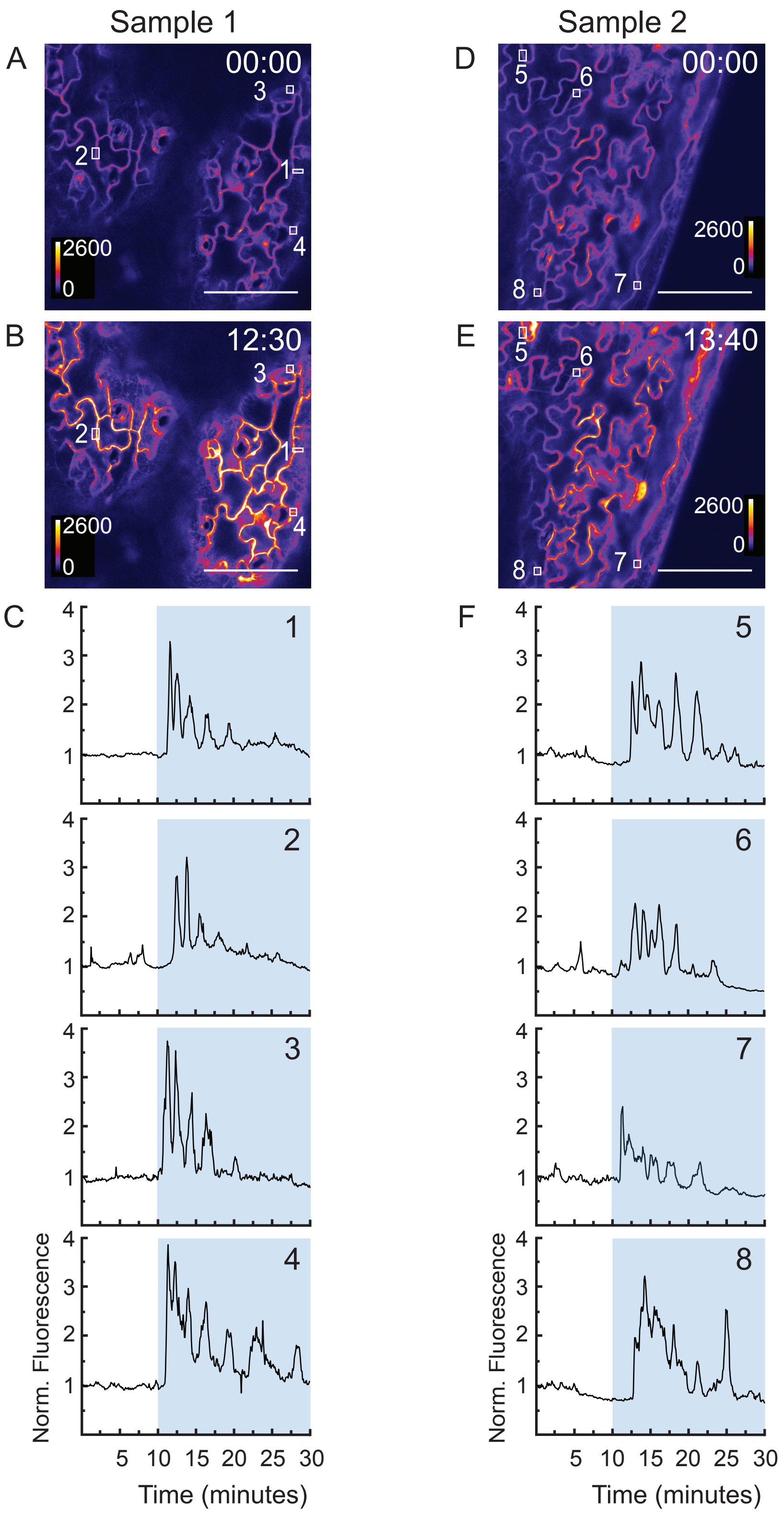

Supplemental Figure 2. Calcium imaging with R-GECO1 using a Hybriwell in response to flg22. Arabidopsis true leaves expressing the intensiometric calcium indicator R-GECO1 were mounted in water in a Hybriwell and imaged before and after treatment with 100 nM flg22. Images of the leaf epidermis were collected every five seconds using a confocal microscope. (A,D) Images of leaf epidermis captured prior to treatment for two independent samples. (B,E) Image of leaf epidermis captured 145 or 215 seconds after treatment, respectively. Fluorescence intensity of the R-GECO1 signal in arbitrary units from 0 to 2600 is shown using a heat map scale. Time stamp indicates minutes:seconds. Scale bar=100 μm. White boxes indicate the regions of interest (ROIs) used to quantitate fluorescence. (C,F) Normalized R-GECO1 fluorescence for individual ROIs. Number in the upper right of each graph indicates the corresponding ROI. Blue background indicates presence of 100 nM flg22 in the Hybriwell.

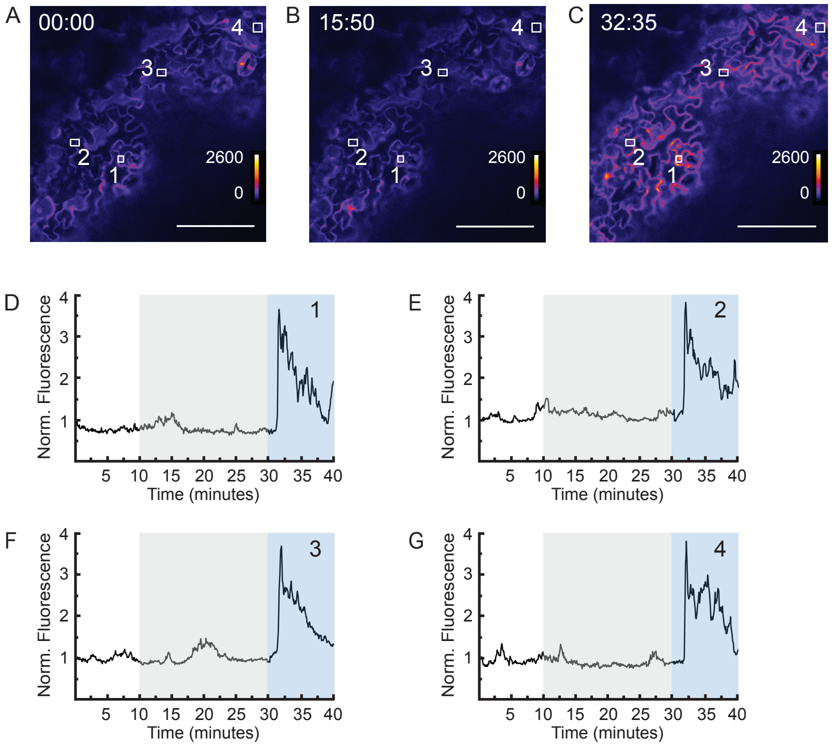

Supplemental Figure 3. Calcium imaging with R-GECO1 using a Hybriwell in response to water. An Arabidopsis true leaf expressing the intensiometric calcium indicator R-GECO1 was mounted in water in a Hybriwell and imaged before and after treatment with pure water, followed later by a secondary treatment of 100 nM flg22. Images of the leaf epidermis were collected every five seconds using a confocal microscope. (A) Leaf epidermis prior to treatment. (B) Leaf epidermis 345 seconds after treatment with pure water. (C) Leaf epidermis 150 seconds after treatment with 100 nM flg22. Fluorescence intensity of the R-GECO1 signal in arbitrary units from 0 to 2600 is shown using a heat map scale. Time stamp indicates minutes:seconds. Scale bar=100 µm. White boxes indicate the regions of interest (ROIs) used to quantitate fluorescence. (D-G) Normalized R-GECO1 fluorescence for individual ROIs. Number in the upper right of each graph indicates the corresponding ROI. Gray background indicates presence of exchanged water in the Hybriwell. Blue background indicates presence of 100 nM flg22 in the Hybriwell.
