# Supplementary material for: A simple microfluidic device for live-cell imaging of Arabidopsis cotyledons, leaves, and seedlings
Source: Biotechniques. Author manuscript; Available in PMC 2021 Nov 15. (PMC8592504; doi:10.2144/btn-2018-0044)
Supplement: Supplementary [file NIHMS1631172-supplement-Supplementary.zip › Supplementary protocol.doc]

Mounting Arabidopsis Samples in a HybriWell^TM^ for Live Cell Imaging

Protocol For:

**A simple microfluidic device for live cell imaging of *Arabidopsis* cotyledons and seedlings**

**Shia Vang^1^, Kati Seitz^2^, and Patrick J. Krysan^1,3*^**

*^1^Horticulture Department, University of Wisconsin-Madison, Madison, WI, USA*

*^2^Laboratory of Genetics, University of Wisconsin-Madison, Madison, WI, USA*

*^3^Genome Center of Wisconsin, University of Wisconsin-Madison, Madison, WI, USA*

*BioTechniques Vol#:pp-pp (Month Year)*

**LEGEND**

* ***HINT***

**REAGENTS**

HBW20 HybriWell^TM^ devices. 20mm Diameter X 0.15mm Depth, 30 µL approximate volume. (Cat. # 611102) (Grace Bio-labs, Bend, OR, USA)

45 mm x 50 mm microscope cover glasses 0.16 – 0.19 mm thick (Fisher Scientific, Waltham, MA, USA) (Cat. # 12-544F)

Murashige and Skoog Basal Salt Mixture (MS) (Sigma-Aldrich, St. Louis, MO, USA) (Cat. # M5524).

Agar, microbiology tested, plant cell culture tested, cell culture tested, powder. (Sigma-Aldrich, St. Louis, MO, USA) (Cat. # A1296).

MES hydrate. (Sigma-Aldrich, St. Louis, MO, USA) (Cat. # M2933).

Filter paper. Whatman Qualitative Circle grade 4, 125 mm diameter. (Sigma-Aldrich, St. Louis, MO, USA) (Cat. # WHA1004125).

Square petri dishes. Fisherbrand 100 x 100 mm. (Fisher Scientific, Waltham, MA, USA) (Cat. # FB0875711A).

3M Micropore tape. (Fisher Scientific, Waltham, MA, USA) (Cat. # 19-027761).

**PROCEDURE**

PLATE ARABIDOPSIS SEEDS

1. Prepare 1.0% (w/v) agar plates containing 0.5X Murashige and Skoog Basal Salt Mixture media with 2 mM MES, pH 5.7, using 100 mm x 100 mm square petri dishes
2. Surface sterilize Arabidopsis seeds by first placing the seeds on a piece of Whatman filter paper in a sterile hood and then dousing them with 95% ethanol until the filter paper is fully saturated with ethanol.
3. Allow the ethanol to evaporate from the filter paper
4. Once the seeds have fully dried (typically 30 to 60 minutes), use a sterile pipet tip to transfer the seeds one-by-one onto the agar growth media.
5. Seal the petri dishes containing the plated seeds using Micropore tape.
6. Place plates in the dark at 4°C for one to three days to stratify the seeds

GERMINATE ARABIDOPSIS SEEDLINGS

7. Place plates in gently reclined vertical orientation so that the emerging roots will grow down the surface of the agar media
8. Incubate under constant light at 18-24°C for three to six days.
9. If using true leaves, move seedlings to soil after six to seven days, and leave under constant light at 18-24°C until leaves reach required age or mass.

* ***HINT****: For imaging seedlings, three days of growth is typically ideal, while detached cotyledons are best at four to six days and true leaves at two weeks.*

MOUNT SAMPLES IN A HYBRIWELL^TM^

10. Place a 45 mm x 50 mm microscope cover glass on a clean lab bench.
11. Place a 5 µl drop of sterile ultrapure water in the center of the cover glass using a micropipette.
12. Remove a cotyledon from a four- to six-day-old seedling growing on a 0.5× MS plate by snipping the petiole with small scissors.
13. Grasp the cotyledon by the petiole using forceps and gently place it on top of the water drop abaxial side up.
14. If the cotyledon is floating on the surface of the water drop, use a micropipette to transfer water from below the cotyledon onto the top surface in order to fully submerge it.
15. Remove excess water using the micropipette, being careful not to damage the cotyledon.
16. Expose the adhesive backing on the HybriWell^TM^ by peeling off the protective covering.
17. Use forceps to gently lower the HybriWell^TM^ down onto the coverglass, positioning it such that the cotyledon is in the center of the HybriWell^TM^ chamber.
18. Use the back of the forceps to gently press the adhesive portion of the HybriWell^TM^ down onto the cover glass to fully seal the chamber, taking care not to press down on the top of the chamber.
19. Fill the chamber with water by rapidly dispensing 300 µl of sterile ultrapure water into one of the ports of the HybriWell^TM^ such that water shoots out the second port during the filling process. Rapidly filling the HybriWell^TM^ using this approach is necessary in order to minimize the chance of a trapping an air bubble in the chamber.
20. If an air bubble is trapped in the chamber, then an additional 300 µl of sterile ultrapure water can be flushed through the HybriWell^TM^ to remove the bubble.
21. Place a 200 µl drop of sterile ultrapure water on top of one of the ports of the HybriWell^TM^ to prevent evaporation of the 30 µl of water within the chamber.
22. Place the HybriWell^TM^ mounted to the cover glass inside a 100 x 100 mm square plastic petri dish and cover to maintain sterility and humidity.
23. Incubate under constant light at 18°-24°C until ready for imaging. Depending on the experiment, samples are typically incubated for 6 to 12 hours prior to imaging to allow the samples to equilibrate to the Hybriwell^TM^.
24. Image samples using an inverted microscope.

* ***HINT****: The same process described above can be used to mount intact seedlings, but in that case the seedlings are transferred to the drop of water on the cover glass by gently lifting them off the growth plate with a sterile plastic 200 µl pipette tip. The same process described above can also be used to mount true leaves, but in that case the leaves are placed on the water drop adaxial side up.*

**RECIPES**

0.5X MS Agar Plates (1 liter)

Murashige and Skoog Basal Salt Mixture (MS)	2.2 g	0.5X

MES Hydrate	0.4 g	2 mM

Agar	10 g	1% (w/v)

adjust pH to 5.7 using KOH

**TROUBLESHOOTING**

Tissue sample is ejected from the HybriWell^TM^ when it is initially filled with water.

Possible cause is that too much force was used when filling the HybriWell^TM^ with	water. To correct this problem reduce the force with which water is pipetted into	the inlet port during initial HybriWell^TM^ filling.
